# A Comparative Cytocompatibility Assessment of Leukocyte-Platelet-Rich Fibrin (L-PRF) and Injectable Platelet-Rich Fibrin (I-PRF) on the Pre-osteoblastic MG-63 Cell Line in Chronic Periodontitis Patients: An In Vitro Study

**DOI:** 10.7759/cureus.48739

**Published:** 2023-11-13

**Authors:** Sheryl Dolly A., Ravishankar PL, Prem Blaisie Rajula, Sai Sri Soury Geddam, Lochini S, Sindhujaa R, Mohamed Rashik

**Affiliations:** 1 Periodontology, SRM Kattankulathur Dental College and Hospital, Chennai, IND

**Keywords:** l-prf, i-prf, regeneration, platelet concentrates, mg-63 cells, cytocompatibility, cell viability

## Abstract

Aim

The aim of the study is to assess the cellular viability of various concentrations of different platelet concentrates on pre-osteoblastic MG-63 cells.

Materials and methods

In this in-vitro experiment, blood samples from 21 individuals with chronic periodontitis were taken and centrifuged according to Choukroun and Miron’s protocol to prepare L-PRF and I-PRF, respectively. The methyl thiazolyl tetrazolium (MTT) test was used to determine the viability of 0%, 1%, 2%, 4%, 8%, 10%, and 20% concentrations of L-PRF and I-PRF on MG-63 cells.

Results

The 20% L-PRF had the lowest percentage of cell viability (90.429±2.06), and the 1% I-PRF had the highest percentage (98.918±0.54), with no statistically significant difference (p>0.05).

Conclusion

According to the findings of the current study, both L-PRF and I-PRF provide favorable outcomes in terms of the viability of MG-63 cells in chronic periodontitis patients that may be utilized for regenerative purposes such as periodontal osseous defects and mucogingival surgeries. Incorporating these platelet concentrates with bone grafts results in enhanced regenerative outcomes.

## Introduction

Periodontitis is characterized by inflammation affecting the tissue supporting the teeth, marked by gradually deteriorating bone and attachment structures. The primary goal of periodontal therapy is to facilitate the regeneration of periodontal tissues, resulting in the formation of new cementum, apposition of new alveolar bone, and the restoration of a functionally aligned periodontal ligament (PDL) [1]. Bone regeneration may not be efficient using conventional treatment methods. Achieving periodontal regeneration necessitates a complex and coordinated series of biological processes, encompassing cell adhesion, migration, proliferation, and differentiation. Numerous attempts have been made for periodontal tissue regeneration, such as bone graft, directed tissue regeneration, tissue graft, and root surface biomodification, such as citric acid, ethylenediaminetetraacetic acid (EDTA), tetracycline, enamel matrix proteins, and hydrogen peroxide [2]. Periodontal regeneration necessitates interactions among osteoblasts, gingival fibroblasts, PDL cells, and epithelial cells [3]. The notion that growth factors (GFs) and platelet-derived cytokines can potentially expedite the healing process and facilitate tissue regeneration has been longstanding in complex clinical situations. The secretion of proteins like fibrin, fibronectin, and vitronectin, which act as both a framework for connective tissue and adhesion molecules that improve the effectiveness of cell migration, is another crucial function of platelets [4]. As a result, it has been suggested that platelets be used as therapeutic agents to enhance tissue repair, particularly in the context of treating periodontal lesions. Platelet concentrates (PC) obtained by centrifugating the patient's blood containing activated platelets become encapsulated within a fibrin matrix scaffold. Since this technique enhances the healing of both soft and hard tissues, PCs have been successfully used in a range of medical and dental fields during the past few decades [5]. In the early 1970s, autologous PC was first used in many dental specialties. They have several benefits besides serving as a GF reservoir, including patient acceptance, cost-effectiveness, and the absence of any ethical concerns, given that they are autologous in origin. However, PC cannot be prepared in patients with platelet malfunction, thrombocytopenia, or those using systemic anticoagulants. The PCs can be classified into first-generation - platelet-rich plasma (PRP), second generation - leukocyte-platelet-rich fibrin (L-PRF), the third generation - advanced-platelet-rich fibrin (A-PRF), and injectable-platelet-rich fibrin (I-PRF), and fourth generation - currently under research (focusing on the tissue engineering triad) [6]. L-PRF, pioneered by Choukroun J et al. [7], comprises both leukocytes and cytokines within a robust fibrin matrix [8]. L-PRF is produced without any anticoagulants, which is unlike platelet-rich plasmas. Along with producing more products, its process is also quicker, cheaper, and less technique-sensitive. Most importantly, the progressive release of GFs that encourage angiogenesis and osteoblastic proliferation and differentiation is made possible by the dense fibrin mesh of L-PRF, which keeps it from disintegrating [9]. By modifying spin centrifugation pressures, I-PRF was developed in 2014. I-PRF, a newly produced platelet concentrate, is enriched with leukocytes and stimulates the regeneration of both soft and hard tissues. Upon application, the human liquid fibrinogen in I-PRF progressively transforms into a clot rich in GFs, continuously releasing them over a period of 10 to 14 days [10]. It remains in liquid form for approximately 15 minutes [11]. Moreover, I-PRF has demonstrated an enhanced capacity to reduce inflammation and combat pathogens, potentially expediting tissue regeneration [12]. In the realm of regenerative dentistry, I-PRF is commonly used as an injectable biomaterial and is utilized in combination with various biomaterials to meet diverse therapeutic requirements [13]. The growing clinical utilization of platelet concentrates like L-PRF and I-PRF to improve bone remodeling around dental implants and teeth justifies the need for further research on these platelet products. MG-63 osteoblast-like cells, which were initially derived from human osteosarcoma, demonstrate several osteoblastic characteristics. These traits include their ability to elevate alkaline phosphatase activity and synthesize osteocalcin in response to 1,25-(OH)2D3 [14]. Thus, this in vitro study aims to compare and evaluate the influence of different concentrations of L-PRF and I-PRF extracts on the cellular viability of pre-osteoblastic MG-63 cells in chronic periodontitis patients.

## Materials and methods

Study design

A randomized single-blinded in vitro trial was done to determine the cytocompatibility of I-PRF and L-PRF on MG-63 cells in patients with chronic periodontitis. The study protocol was discussed and accepted by the SRM Kattankulathur Dental College and Hospital Institutional Ethical Committee Review Board (SRMIEC-ST0923-592). This study population was recruited from the dental OPD of the Department of Periodontology, SRM Kattankulathur Dental College and Hospital. Blood sample collection was conducted for the study over a duration of one month.

Inclusion and exclusion criteria

Twenty-one volunteers (11 females and 10 males), aged 30-55 years and with either generalized or localized chronic periodontitis, were included in the study. All participants had a probing pocket depth of ≥5mm and were systemically healthy. Those with a history of diabetes or who had taken medications such as aspirin in the past two weeks were excluded.

Preparation of L-PRF and I-PRF extracts

After explaining the study's methodology, consent forms were obtained from the participants. Subsequently, 10 mL of blood was drawn from the antecubital veins of the 21 participants under sterile conditions. This blood was then placed in 10-mL dry glass-coated plastic tubes for the preparation of L-PRF and in plastic tubes specifically for the preparation of I-PRF (Biopro iPRF Tube, Alchem Diagnostics, Coimbatore, Tamil Nadu). To prepare the L-PRF and I-PRF, the tubes were centrifuged according to the procedures outlined by Dohan DM et al. [15] (2700 RPM, 12 minutes) and Miron RJ et al. [16] (700 revolutions per minute, 3 minutes), respectively, with no anticoagulant added. After centrifugation, the tubes were transferred to a biosafety cabinet. Here, the L-PRF fibrin clots were held with forceps and carefully severed from the red corpuscle layer using scissors. A 2 mL syringe was used to immediately collect the I-PRF that was formed at the top layer. The cytocompatibility of these platelet concentrates was further evaluated.

Preparation of cells

The MG-63 cell line, which shares similarities with human osteoblasts, was acquired from the National Centre for Cell Science (NCCS) in Pune, India. These cells were grown in T255 culture flasks using Dulbecco's Modified Eagle Medium (DMEM) enriched with 10% fetal bovine serum (FBS). Upon reaching confluence, the cells were dissociated using a Trypsin-EDTA solution.

Cytocompatibility assay

To assess the cytocompatibility of L-PRF and I-PRF, their induction at different concentrations (0%, 1%, 2%, 4%, 8%, 10%, and 20%) was evaluated on MG-63 osteoblast-like cells over 24 hours using an MTT assay, as previously described by Koka P et al., 2018 [17]. Briefly, MG-63 cells were incubated with varying concentrations of L-PRF and I-PRF and then seeded on a 96-well culture plate for 24 hours. To assess cell viability after this incubation period, 10 𝜇l of a stock MTT dye solution (10 mg/ml) was added to each well. The plate was then incubated again at 37 °C for 4 hours. Subsequently, 100 μl of dimethylsulfoxide (DMSO) was introduced to each well to dissolve the formazan crystals, and the absorbance was measured at 570 nm using Synergy Hybrid Multi-Mode Reader (BioTek, Winooski, VT, US). The percentage of cell viability was determined using the following formula:

Cell viability % = OD (test sample) - OD (blank)/ OD(PC) - OD (blank) X 100

## Results

**Figure 1:**
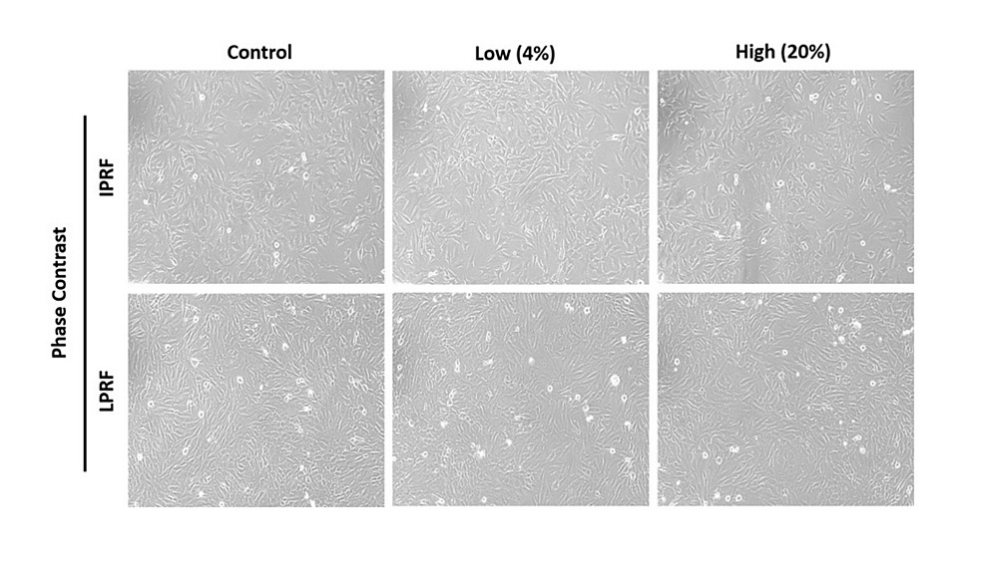
Morphological evaluation using phase contrast microscopy: The biocompatibility of L-PRF and I-PRF, induced by both low (4%) and high (20%) concentrations, was evaluated in comparison with a control group on MG-63 osteoblast-like cells. This assessment was conducted using both phase contrast and fluorescent microscopy. Magnification: 20X. L-PRF: Leukocyte-Platelet-Rich Fibrin, I-PRF: Injectable Platelet-Rich Fibrin.

The data were statistically analyzed using the program SPSS version 23 (IBM Corp., Armonk, NY, USA). One sample t-tests were used for intragroup comparisons of cell viability on osteoblastic cell line at various concentrations of L-PRF and I-PRF, and paired sample t-tests were used for intergroup comparisons of cell viability on MG-63 cells between L-PRF and I-PRF at various concentrations. We utilized the MTT assay to evaluate the influence of different concentrations (0%, 1%, 2%, 4%, 8%, 10%, and 20%) of L-PRF and I-PRF on cell viability. Figure 1 illustrates the morphological assessment through phase-contrast microscopy. For both experimental groups, cell viability was set at 100% when using 0% L-PRF and I-PRF.

The intragroup comparison of L-PRF presented in Table 1 revealed the maximum cell viability percentage in 1% (97.575±1.10) which further decreased with the increase in concentration such as 2% (96.425±1.33), 4% (95.134±1.81), 8% (93.254±1.83), 10% (92.050±1.93), 20% (90.429±2.06). Similarly, the intragroup comparison of I-PRF presented in Table 2 revealed the maximum cell viability percentage in 1%(98.918±0.54), which also further decreased with the increase in concentration such as 2% (97.780±0.73), 4% (95.341±1.19), 10 (94.236±1.32), and 20% (93.107±1.27). In each experimental group, the percentage of cells at different concentrations of I-PRF and L-PRF significantly reduced at 24 hours (p< 0.000).

**Table 1:** Intragroup comparison of mean viability of MG-63 cells at various concentrations of L-PRF in chronic periodontitis patients at 24 hours. L-PRF: Leukocyte-Platelet-Rich Fibrin.

Different concentrations of L-PRF	Test Value = 0					
	t	df	Sig. (2-tailed)	Mean Difference	95% Confidence Interval of the Difference	
					Lower	Upper
1%	404.956	20	0.000	97.57503	97.0724	98.0776
2%	332.092	20	0.000	96.42565	95.8200	97.0313
4%	239.820	20	0.000	95.13471	94.3072	95.9622
8%	232.564	20	0.000	93.25430	92.4179	94.0907
10%	217.845	20	0.000	92.05005	91.1686	92.9315
20%	200.538	20	0.000	90.42939	89.4888	91.3700

**Table 2:** Intragroup comparison of mean viability of MG-63 cells at various concentrations of I-PRF in chronic periodontitis patients at 24 hours. I-PRF: Injectable Platelet-Rich Fibrin.

Different concentrations of I-PRF	Test Value = 0					
	t	df	Sig. (2-tailed)	Mean Difference	95% Confidence Interval of the Difference	
					Lower	Upper
1%	825.723	20	0.000	98.91800	98.6681	99.1679
2%	606.704	20	0.000	97.78797	97.4518	98.1242
4%	500.912	20	0.000	96.51428	96.1124	96.9162
8%	365.164	20	0.000	95.34162	94.7970	95.8863
10%	381.324	20	0.000	94.23631	93.7208	94.7518
20%	334.139	20	0.000	93.10724	92.5260	93.6885

**Figure 2:**
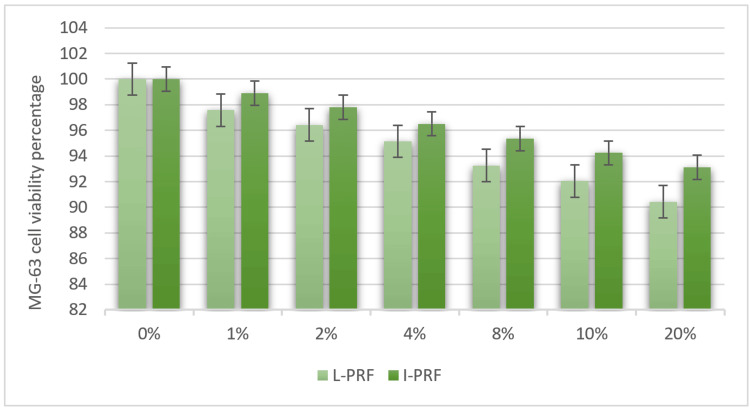
Viability assessment of the osteoblastic cell line (MG-63) at various experimental concentrations in chronic periodontitis patients. L-PRF: Leukocyte-Platelet-Rich Fibrin; I-PRF: Injectable Platelet-Rich Fibrin.

In the comparison between the L-PRF group and the I-PRF group presented in Table 3, it was observed that the I-PRF group exhibited a higher average percentage of cell viability after 24 hours. Consequently, the intergroup assessment of L-PRF and I-PRF effects on MG-63 cell viability at 24 hours was not statistically significant. The highest percentage of cell viability was observed in 1% I-PRF (98.918 ±0.54), and the lowest percentage of cell viability was observed in 20% L-PRF (90.429 ± 2.06) with no significance. Figure 2 depicts the graphical representation of the viability assessment conducted on the osteoblastic cell line (MG-63) across various experimental concentrations in individuals with chronic periodontitis.

**Table 3:** Intergroup comparison of mean viability of MG-63 cells in different experimental groups (L-PRF and I-PRF) among chronic periodontitis patients after 24 hours. L-PRF: Leukocyte-Platelet-Rich Fibrin; I-PRF: Injectable Platelet-Rich Fibrin.

Concentrations	Groups	Mean	N	Std. deviation	Std. error mean	Correlation	Significance
1%	L-PRF	97.5750	21	1.10418	.24095	-.293	.198
	I-PRF	98.9180	21	.54897	.11980		
2%	L-PRF	96.4257	21	1.33059	.29036	. 030	. 898
	I-PRF	97.7880	21	.73862	.16118		
4%	L-PRF	95.1347	21	1.81787	.39669	.008	.974
	I-PRF	96.5143	21	.88296	.19268		
8%	L-PRF	93.2543	21	1.83754	.40098	.114	.623
	I-PRF	95.3416	21	1.19648	.26109		
10%	L-PRF	92.0501	21	1.93636	.42255	-.006	.979
	I-PRF	94.2363	21	1.13249	.24713		
20%	L-PRF	90.4294	21	2.06644	.45093	-.001	.998
	I-PRF	93.1072	21	1.27693	.27865		

In general, the I-PRF group displayed higher percentages of cell viability compared to the L-PRF group, and this was consistent with a decline in viability as the concentration increased for both the I-PRF and L-PRF groups difference(p>0.05).

## Discussion

PCs have become cutting-edge autologous blood products that improve tissue regeneration and repair in regenerative medicine. The presence of platelets in these is essential for bone remodeling, homeostasis, facilitating the production of fibrin clots, and secreting substances that promote angiogenesis. Leukocytes have a crucial role in infection prevention by engulfing and eliminating debris and dead tissues through the process of phagocytosis [18]. Macrophages have a specific role in producing GFs such as Platelet-Derived Growth Factor (PDGF), Transforming Growth Factor (TGF), and Vascular Endothelial Growth Factor (VEGF). They are also a source of chemotactic substances required to stimulate angiogenesis [19]. These GFs and hormones influence cell differentiation and growth regulation, which regulates bone regeneration [20]. Moreover, in a systematic analysis of randomized controlled trials conducted by Chen J et al. [21], it was found that platelet-rich products facilitate the process of wound healing. The results from this current test indicate that the percentage of cell viability in the I-PRF group, across all tested concentrations, was higher than that in the L-PRF group at the 24-hour mark. However, this difference did not reach statistical significance. In the context of clinical applications, assessing the clinical significance and practical implications of these findings becomes crucial. While statistical significance provides valuable information about the likelihood that the observed results are not due to chance, clinical significance assesses whether these findings have practical importance in real-world scenarios. Further research and clinical trials are warranted to determine the practical implications of these differences in cell viability for regenerative purposes. The outcomes suggest that these products could be used to treat bone abnormalities and that I-PRF can promote the functional differentiation of the osteoblast-like cells than the other PCs. This may highlight the variations in GF levels between these two platelet products, which are controlled by the duration and speed of centrifugation. Choukroun J et al. [11] noted that a reduction in relative centrifugal force during research led to a notable increase in the quality and quantity of platelets, leukocytes, fibrin matrix, and GFs in L-PRF. In contrast, I-PRF demonstrated elevated levels of collagen 1, PDGF, TGF, and enhanced fibroblast migration, which was reported by Miron RJ et al. [16].

Furthermore, our study verified that the impact of L-PRF on the viability of MG-63 cells is dose-dependent. This observation aligns with the findings of Esmaeilnejad A et al. [22], who reported that cell viability in response to L-PRF at the tested concentrations (0.5%, 1%, 10%, and 20%) decreased at 24 hours and exhibited a significant increase at 72 hours. It is worth noting that our study, in contrast, focused solely on a 24-hour timeframe. The authors also addressed the influence of time on the proliferation rate, highlighting a notably higher rate during the 72 hours in contrast to the 24-hour timeframe. Interestingly, these findings contradict the study conducted by Li X et al. [23], which affirmed that prolonged exposure to higher concentrations of L-PRF exudates significantly enhances PDL cell proliferation. According to our study, the cell viability of I-PRF might be due to their concentration, the GFs present in them, and the ability of I-PRF to enhance early cell differentiation, as stated by Kosmidis K et al. [24]. Wang X et al.'s initial research looked at how I-PRF culture affected primary human osteoblasts' ability to proliferate, differentiate, mineralize, adhere, and migrate [25]. As per the researchers, in comparison to PRP, I-PRF induced a three-fold rise in the migration of human osteoblasts. Furthermore, it was observed that on the third and fifth days, I-PRF led to a significant increase in proliferation compared to PRP, with no notable differences in terms of cell attachment. An investigation into how I-PRF affects gingival mesenchymal stem cell proliferation and osteogenic differentiation was done by Iozon S et al. [26]. According to their research, cell proliferation was greatly decreased in the culture with 10% I-PRF after seven days and significantly boosted in the culture with 5% I-PRF. The use of I-PRF in cultures of gingival mesenchymal stem cells was associated with decreased expression of all osteogenic genes. Fernández-Medina T et al. [27] observed detrimental effects on cell viability, metabolic activity, and migration when I-PRF concentration surpassed 60%. In our current investigation, we observed a significant difference within the MG-63 cell group for patients with chronic periodontitis, both in the L-PRF and I-PRF groups. The study's strengths lie in its controlled in vitro experimental design, which enables precise evaluation of cellular viability in response to PCs, reducing confounding factors. Additionally, the research addresses a clinically relevant topic related to periodontitis and regenerative dentistry, offering practical implications for clinical practice. Using the established and reliable MTT assay further enhances the study's credibility. Combining these PRFs appears to offer promise for situations requiring bone grafting due to their positive impact on cell viability in pre-osteoblastic cells, as evidenced in this study. Additionally, PRFs can potentially preserve bioactive materials and serve as scaffolds. However, it is crucial to acknowledge the potential adverse effects on cell growth that this combination may entail.

Limitations

Considering the limitations of our current study, it is essential to acknowledge that our study involved 21 individuals with chronic periodontitis, which may be considered relatively small in the context of clinical research. It is worth noting that our study might represent a preliminary investigation into the effects of I-PRF on cell viability. Often, early research serves as a foundation for more comprehensive studies. These findings could prompt further research with larger sample sizes to potentially yield more robust and generalizable results. Another important limitation is that our study focused on a 24-hour timeframe, which offers valuable insights into short-term effects. However, it is essential to recognize that this limited scope may not fully capture the longer-term outcomes. In light of the identified limitations of our study, to facilitate the advancement of knowledge in this field, we propose several areas for further investigation, such as the distinct roles of GFs (e.g., PDGF, TGF, and VEGF) and their optimal concentrations within PCs to optimize their regenerative potential. Furthermore, it is prudent to consider a broader spectrum of cellular responses, encompassing not only cell viability but also proliferation and mineralization ability, to gain a more comprehensive understanding of regenerative mechanisms. Extending the study duration and incorporating assessments at multiple time points is also recommended to comprehensively elucidate longer-term effects.

## Conclusions

Based on the results of the current study, both L-PRF and I-PRF show positive results regarding the viability of MG-63 cells, which can be used for regenerative purposes. Also, I-PRF showed better viability than L-PRF.
